# Editorial: Functional Genomics in Fruit Trees: From ‘Omics to Sustainable Biotechnologies

**DOI:** 10.3389/fpls.2021.729714

**Published:** 2021-07-26

**Authors:** Concetta Licciardello, Irene Perrone, Giorgio Gambino, Manuel Talon, Riccardo Velasco

**Affiliations:** ^1^CREA—Research Centre for Olive, Fruit and Citrus Crops, Acireale, Italy; ^2^National Research Council (CNR), Institute for Sustainable Plant Protection, Torino, Italy; ^3^Instituto Valenciano de Investigaciones Agrarias (IVIA), Centro de Genómica, Valencia, Spain; ^4^CREA—Research Centre for Viticulture and Enology, Conegliano, Italy

**Keywords:** woody plants, transcriptomics, metabolomics, resequencing, new breeding techniques, genetic diversity, fruit quality, plant pathogen

This Research Topic of Frontiers in Plant Science collects 8 manuscripts, focused on fruit crops of high commercial interest, such as bayberry, blueberry, pear, grapevine, citrus, and walnut. The breadth of solutions and approaches that omics offer us today, and the applications and perspectives that are looming over a short time horizon, are illustrated with a particular focus on fruit qualitative traits, related to firmness and post-harvest (Cappai et al.), ripening (Honaas et al.), and lignin accumulation (Cao et al.), on improving knowledge on secondary metabolites, such as phenolic compounds (Saxe et al.), as well as on investigating earliest responses to pathogens (Wei et al.). Moreover, the Research Topic illustrates a technological application path from whole-genome sequencing (Wu et al.) to the resequencing (Tanaka et al.), to achieve the ultimate objective of modulating genes using the New Breeding Techniques (Salonia et al.) (Figure 1).

**Figure 1:**
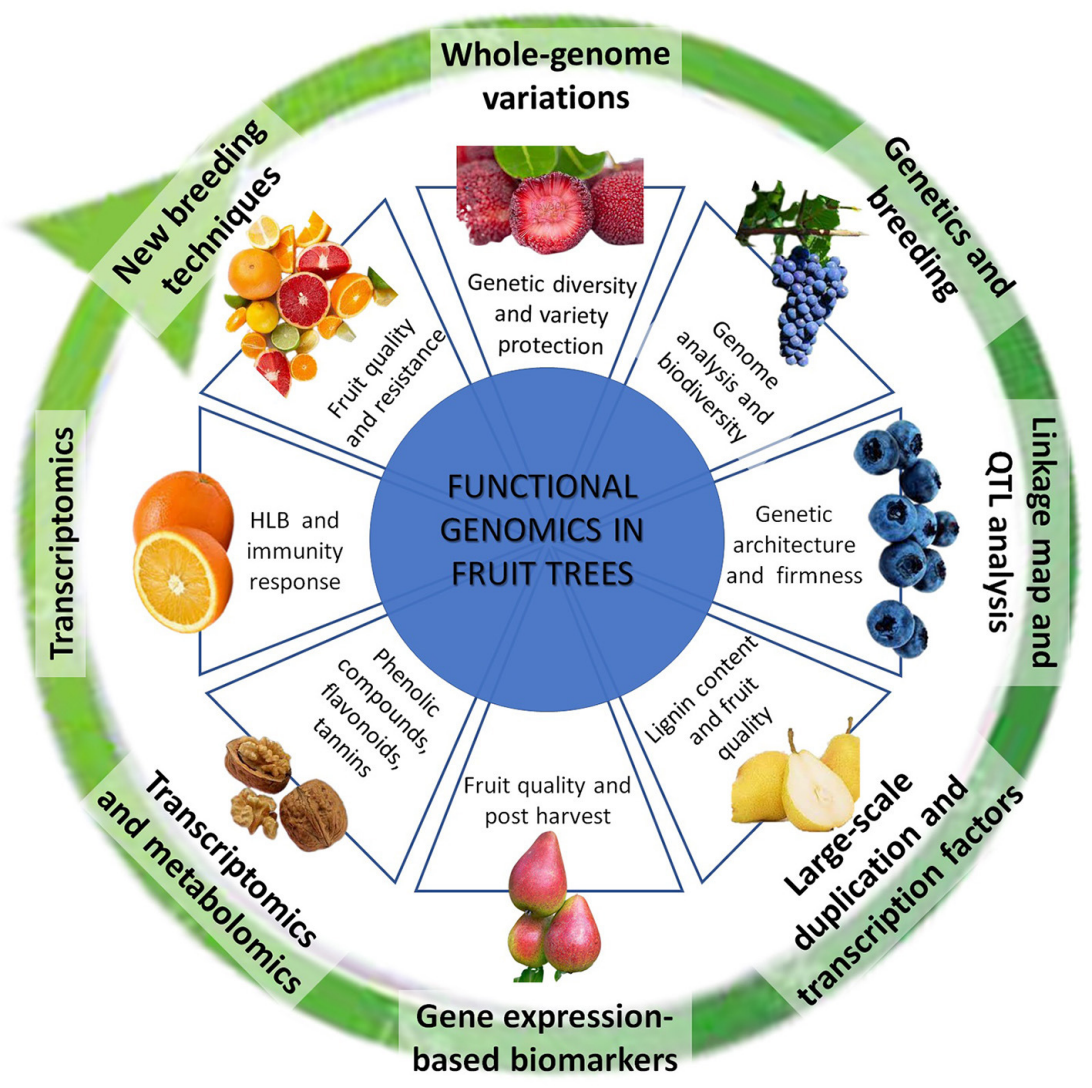
Methods and applications of Functional Genomics in fruits trees. The figure summarizes the main methodologies reported in the manuscripts collected in this Research Topic, represented as a technologically evaluative route that reach the use of new breeding techniques, starting from the knowledge of genome. The plant species and the application scope are punctually showed.

Fruit trees are economically important species and, as long-living plants, represent an important challenge to understand adaptation to environmental stresses. Detailed functional analyses are often difficult in woody species, because of their biological features and the recalcitrance of some species to plant transformation and/or regeneration, impediments that greatly limit the use of standard genetic and biotechnological approaches for functional genomics studies or plant breeding.

Specifically, fruit tree species are difficult to study at genetic and molecular levels (1) because of their perennial nature, (2) the limited information on gene identity and function, and genetic markers directly associated to the control of a character, (3) the unavailability of well-defined molecular genetic linkage maps and (4) the poor development of mapping population and map-based studies, that are generally rather complex in woody plants.

Over the last decade, the sequencing of several genomes, coupled with rapid advances in bioinformatics, provided powerful tools for detailed molecular studies on crop plants other than traditional model species. The availability of sequencing data is only a starting point since bioinformatics approaches are not sufficient to define gene roles. To deepen this knowledge, it is necessary to understand how thousands of genes can interact each other to define the structure of a plant and how the metabolic pathways in which they are involved contribute to plant development and adaptation to the environment.

The impressive possibilities that genome sequencing linked with intraspecies variability may provide, are represented by the manuscript contributed by Wu et al., reporting on an exhaustive and robust study of the red bayberry genome. The authors explore the genetic diversity of this species in China and study the distribution of whole-genome variations (SNPs, indels and structural variations) in around fifty accessions. By selecting specific regions of the genome, the authors also identify the true genotypes from the whole pool of current varieties, separating them from sport mutations or simply synonyms. Taken together, the results provide a much more complete view of the genetic diversity of this species and reveal new insights about its domestication processes. From an applied point of view, the design of a DNA fingerprinting system constitutes a tool of maximum utility in molecular breeding and variety protection.

A whole-genome resequencing together with a bioinformatics analysis elucidated genomic variations of a Japanese indigenous wine grape “Koshu” used as a table and processing, respect to table grape white cultivar “Thompson Seedless” (“Sultanina”) and European black one “Tannat.” Tanaka et al. developed a deep analysis demonstrating that “Koshu” is phylogenetically distinct from the other two grape cultivars, as also supported by the presence of small and structural variations. In particular, a high heterozygosity was observed in the chromosome 7 where clusters of resistance genes are sited. Moreover, “Koshu” genomic information, supported by transcriptional data, contribute to explain differences in term of higher polyphenol content in the berries and wine. The Japanese variety represents an interesting genetic resource that can be used for improving the wine quality, through application of genome-wide association studies, genomic selection, and marker-associated selection.

Although fundamental for the breeding of globally important fruit crops, genetic studies on polyploid species are methodologically demanding, as evidenced by the work of Cappai et al. The authors describe the generation of a high-resolution genetic linkage map for the autotetraploid crop Highbush blueberry (*Vaccinium corymbosum* L.), followed by QTL mapping to reveal the genetic architecture of the firmness trait. From an applied point of view, the emerging knowledge about traits related to machine harvestability, such as firmness, is valuable. The development of cultivars suitable for mechanical harvesting is extremely important to the blueberry industry, since cost due to the laborious hand-picking process is very high. Therefore, proposed QTLs represent a strategic source for putative gene investigation to be used for future molecular breeding studies on machine harvesting.

The availability of many genomic data allowed the genome-wide identification of BZR transcription factors (TFs) in five Rosaceae species by Cao et al. These TFs are crucial regulators of Brassinosteroid (BR) synthesis and play essential roles in plant growth and environmental stimuli. BZR members in the pear genome showed large-scale duplication events during evolution. In particular, three PbBZR genes in Chinese pear (*Pyrus bretschneideri*) were identified as nuclear proteins and the suppression of PbBZR1 by VIGS led to a significant increase in the lignin content of *P. bretschneideri* fruits. PbBZR1 is a transcriptional repressor of lignin biosynthetic genes and considering that the lignin content can hinder the value and quality of commercial fruit, PbBZRs resulted important target genes for functional studies and molecular breeding to improve *P. bretschneideri* fruit quality.

Proper ripening capacity is a critical factor for postharvest management of European pear because fruit must achieve specific quality features to meet consumer expectations. The work of Honaas et al. aims to provide insights into the molecular mechanisms that control “d'Anjou” pear (*Pyrus communis* L.) ripening and specifically to provide gene expression-based biomarkers that might be useful to producers. Combining a transcriptomic approach with bioinformatic tools to assess gene co-expression networks, the authors describe the expression patterns of gene modules associated with fruit quality and differences in maturity at harvest. Genes related to photosynthesis, alpha-farnesene biosynthesis and epigenetic machinery revealed to be essential in improving pear quality.

Functional genomic approaches have been adopted by Saxe et al. for the characterization of JrGGT1 and JrGGT2 genes isolated from *Juglans regia* (English Walnut). Transcriptomic and metabolic analysis in walnut and transgenic overexpression in tobacco demonstrated that, despite the high sequence similarity at the nucleotide and amino acid levels, the two genes showed different correlations within the secondary metabolism in plant. Both JrGGTs are UDP84A type glycosyltransferases and are involved in the metabolism of shikimic acid and phenylpropanoid. However, JrGGT1 is a regulator of the metabolism of hydroxybenzoic and hydroxycinnamic acids, while JrGGT2 appears to be more involved in the metabolism of specific flavonoids. These glycosyltransferases definitely play an important role in the metabolism of phenolic acids, flavonoids, and tannins in walnut as well as in other woody species.

A transcriptomic approach was also reported to investigate a very early response (1 day post infection (dpi) and the overreacted defense reaction at 5 dpi) of Valencia orange (*Citrus sinensis* L. Osbeck) to Huanglongbin (HLB), the most devastating citrus disease, caused by the bacterium *Candidatus Liberibacter asiaticus* (CaLas) and its vector Asian citrus psyllid (Wei et al.). The contribution of this study to the today knowledge is that CaLas not only uses virulence strategies to overcome the host cell immunity by switch-off genes of the signaling pathways; CaLas is also able to modify host cellular metabolic pathways to obtain necessary energy and accelerate the replication process. This study contributes to give new foundation for further development of HLB-resistant germplasm that, through genetic manipulation, make feasible and reliable solutions for a new sustainable citrus culture.

All these studies represent the prerequisite to make applicable the New Plant Breeding Techniques (NPBTs), such as genome editing and cisgenesis. Salonia et al. reviewed the state of art of NPBTs in *Citrus*, discussing the main traits (apomixis, high heterozygosity, long juvenility phase, poor regenerative ability) that make this species so difficult to be managed. The limited knowledge of causative genes (one gene, one function) contributes to explain the limited examples of the modern biotechnologies application in this species. The use of explants from young *in vitro* plantlets instead of mature plants, the optimization of current regeneration protocols to overcome the recalcitrance of some genotypes, the need of marker-free systems and shortening the long juvenility phase are also discussed. Actually, cisgenesis and genome editing are being used to make resistant to several devasting diseases, such as HLB and Citrus canker, and to improve citrus fruit quality making them healthier and seedlessness.

Overall, there is no doubt that the advent of omics technologies over the past two decades is transforming the way we currently explore, understand and interpret the smallest aspect of each and every biological discipline. The irruption of the state-of-the-art genomic techniques and protocols has been providential in the advancement of knowledge in harsh disciplines that are difficult to approach, such as the study of the physiological behavior of fruit crops, which in essence are far from the classical models traditionally used in plant biology. In this sense, the papers collected in the Research Topic *Functional Genomics in Fruit Trees: from ‘Omics to Sustainable Biotechnologies* provide a representative and valuable summary of the current applications of the omics technologies in woody plants.

## Author Contributions

All authors listed have made a substantial, direct and intellectual contribution to the work, and approved it for publication.

## Conflict of Interest

The authors declare that the research was conducted in the absence of any commercial or financial relationships that could be construed as a potential conflict of interest.

## Publisher's Note

All claims expressed in this article are solely those of the authors and do not necessarily represent those of their affiliated organizations, or those of the publisher, the editors and the reviewers. Any product that may be evaluated in this article, or claim that may be made by its manufacturer, is not guaranteed or endorsed by the publisher.

